# Prognostic roles of Notch receptor mRNA expression in human ovarian cancer

**DOI:** 10.18632/oncotarget.16387

**Published:** 2017-03-20

**Authors:** Chuan Chen, Xiaojiao Wang, Shunping Huang, Lin Wang, Lili Han, Songtao Yu

**Affiliations:** ^1^ Cancer Center, Daping Hospital and Research Institute of Surgery, Third Military Medical University, Chongqing 400042, P.R. China; ^2^ Department of Oncology, Southwest Hospital, Third Military Medical University, Chongqing 400038, P. R. China; ^3^ Department of Oncology, The Second Affiliated Hospital of Chongqing Medical University, Chongqing 400010, P. R. China; ^4^ Department of Gynecology, People' s Hospital of Xinjiang Uyhgur Autonomous Region, Urumqi, Xinjiang 830001, P. R. China

**Keywords:** Notch receptor, mRNA expression, KM plotter, hazard ratio, prognosis

## Abstract

Aberrant activation of Notch signaling pathway has been correlated with high grade ovarian carcinoma and carcinogenesis. However, the predictive and prognostic values of Notch signaling pathway in ovarian cancer patients remains unclear. We utilize “The Kaplan-Meier plotter” (KM plotter) background database to access the prognostic values including overall survival (OS), progression-free survival (PFS), as well as post-progression survival (PPS) of four Notch receptor mRNA expression in ovarian cancer patients. *Notch1* mRNA high expression was not correlated with OS, PFS and PPS for all ovarian cancer patients, but significantly correlated with poor PFS in *TP53* wild type and favorite PFS in *TP53* mutation type ovarian cancer patients. *Notch2* mRNA high expression was significantly correlated with poor PFS for all ovarian cancer patients, especially in grade II patients. *Notch3* mRNA high expression was significantly correlated with favorite PFS for all ovarian cancer patients. *Notch4* mRNA high expression was significantly correlated with favorite OS, but not PFS and PPS for all ovarian cancer patients. The results strongly support that there are distinct prognostic values of four Notch receptor mRNA expression in ovarian cancer patients.

## INTRODUCTION

Cancer of the ovary is not common, but it ranks fourth as the cause of cancer deaths and causes more deaths than other female reproductive cancers in women [1–2]. Most ovarian tumors often initiated from ovarian surface epithelial (OSE) cells, thus have epithelial origins [3]. Although the improvement in early diagnosis, surgery, various operations for the radical cure, chemotherapy, targeted therapeutic treatment and the emerging immunotherapy, most of the cancer patients would experience recurrent disease following first-line therapy [4–5]. Therefore, the study on the molecular mechanisms of carcinogenesis and identification of differential diagnostic, prognostic marker is still needed.

The Notch signaling pathway that regulates the maintenance of stem cells and controls cell-fate decisions is an evolutionarily conserved system [6–7]. Deregulated expression of four Notch receptors and their ligands has been observed in several human malignancies including ovarian cancer [8–11]. The aberrant activation of Notch signaling pathway plays the imperative roles in ovarian cancer carcinogenesis and chemoresistance of ovarian cancer patients [12–17]. Recently, a number of studies also demonstrated that Notch signaling pathway, especially Notch1 is important for maintaining cancer stem cells in ovarian cancer [18–20]. DAPT, a γ-secretase inhibitor, which reduces gamma-secretase in Notch1 signaling pathway was reported as a highly promising novel therapeutic drug candidate for ovarian cancer patient [21]. LY900009, a first-in-human phase I study of the oral Notch inhibitor was also reported in patients with advanced cancer including ovarian cancer [22]. MK-0752 is another novel γ-secretase inhibitor, which is evaluated in clinical trial for treatment of several types of cancer including ovarian cancer [23]. However, at mRNA level, the predictive roles of individual Notch receptors in ovarian cancer patients remain unknown. In this study, we accessed the predictive roles of Notch receptor mRNA expression in human ovarian cancer patients.

The “Kaplan-Meier plotter” (KM plotter) was capable of assessing the effect of 54,675 genes on survival of 1,648 ovarian cancer patients (http://kmplot.com/analysis/) [24]. KM plotter [25] (http://kmplot.com/analysis/index.php?p=service&cancer=ovar), handled by a PostgreSQL server, which integrates gene expression and clinical data simultaneously. KM plotter was established using gene expression data and survival information [25]. Until now, several genes, such as ALDH1, ITIH5, CK2, GREB1 have been identified and validated by KM plotter in lung cancer [26–9], breast cancer [29–39], as well as in ovarian cancer [29, 40–41]. In this study, we took advantage of KM plotter and accessed the prognostic roles of four Notch receptors in 1,648 ovarian cancer patients.

## RESULTS

Notch receptors include Notch1~4 family members. All Notch receptors can be found Kaplan-Meier OS, PFS, as well as PPS information in the KM plotter database.

**Figure 1:**
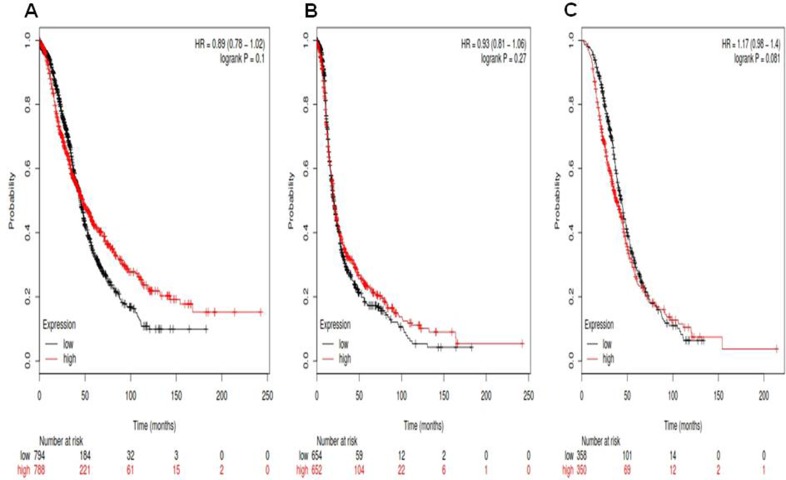
For Notch1, its Affymetrix ID is 218902_at (**A**) OS curves are plotted for ovarian cancer patients (*n* = 1,582). (**B**) PFS curves are plotted for ovarian cancer patients (*n* = 1,306). (**C**) PPS curves are plotted for ovarian cancer patients (*n* = 708).

For Notch1, its Affymetrix ID is 218902_at. OS curves are plotted for ovarian cancer patients (*n* = 1,582) (Figure 1A), PFS curves are plotted for ovarian cancer patients (*n* = 1,306) (Figure 1B) and PPS curves are plotted for ovarian cancer patients (*n* = 708) (Figure 1C). *Notch1* mRNA high expression was not correlated to OS for all ovarian cancer patients followed for 20 years, HR 0.89 (0.78–1.02), *p* = 0.1. *Notch1* mRNA high expression was also not correlated to PFS ovarian cancer patients, 0.93 (0.81–1.06), *p* = 0.27, as well as PPS in ovarian cancer patients, HR 1.17 (0.98–1.4), *p* = 0.081.

**Figure 2:**
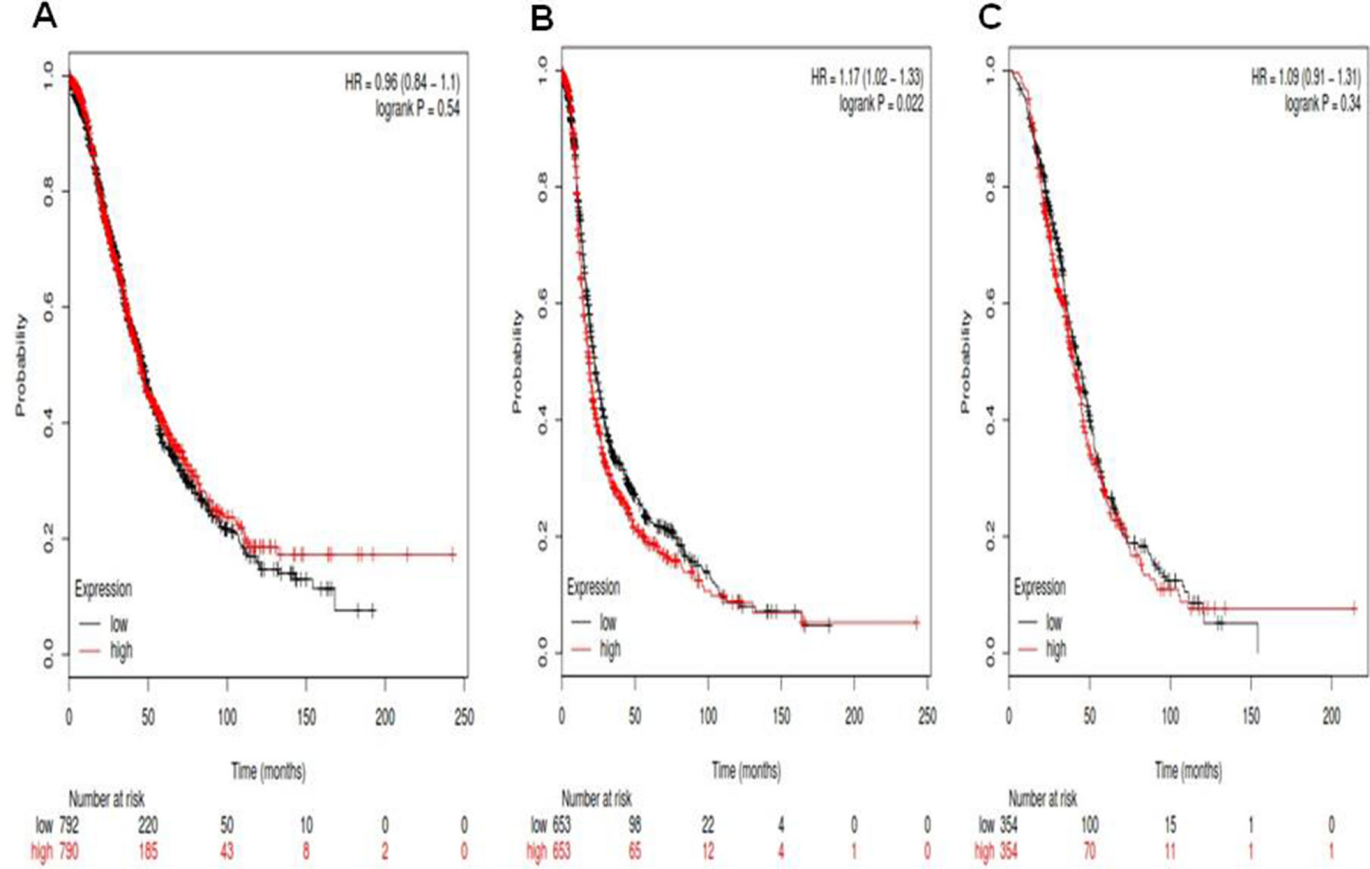
For Notch2, its Affymetrix ID is 210756_s_at (**A**) OS curves are plotted for all ovarian cancer patients (*n* = 1,582). (**B**) PFS curves are plotted for ovarian cancer patients (*n* = 1,306). (**C**) PPS curves are plotted for ovarian cancer patients (*n* = 708).

For Notch2, its Affymetrix ID is 210756_s_at. *Notch2* mRNA high expression was not correlated to OS for all ovarian cancer patients HR, 0.96 (0.84–1.1), *p* = 0.54 (Figure 2A). However, *Notch2* mRNA high expression was significantly correlated to poor PFS for all ovarian cancer patients, HR 1.17 (1.02–1.33), *p* = 0.022 (Figure 2B). *Notch2* mRNA high expression was not correlated to PPS in ovarian cancer patients, HR 1.09 (0.91–1.31), *p* = 0.34 (Figure 2C).

**Figure 3:**
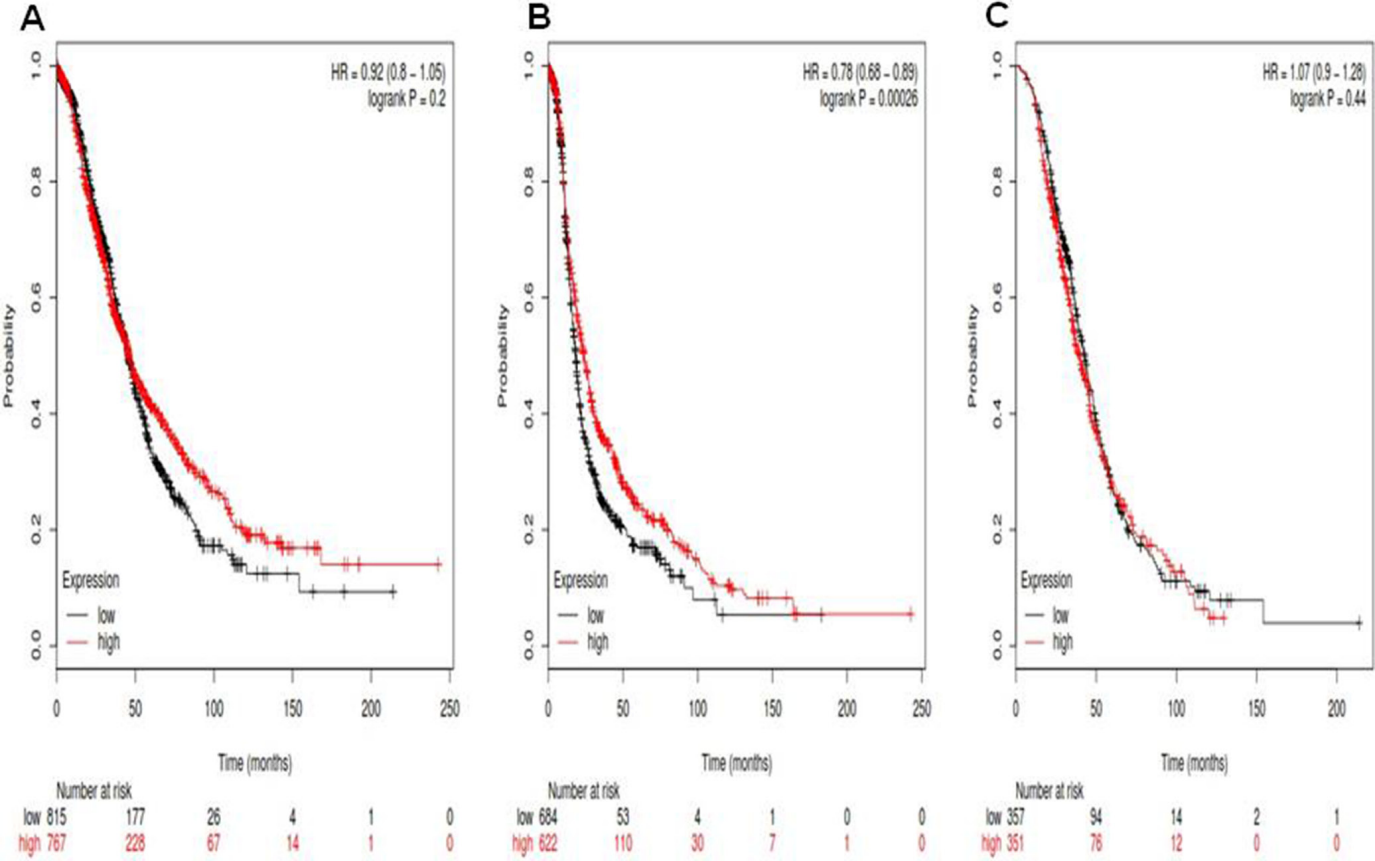
For Notch3, its Affymetrix ID is 203237_at (**A**) OS curves are plotted for all ovarian cancer patients (*n* = 1,582). (**B**) PFS curves are plotted for ovarian cancer patients (*n* = 1,306). (**C**) PPS curves are plotted for ovarian cancer patients (*n* = 708).

For Notch3, its Affymetrix ID is 203237_at. *Notch3* mRNA high expression was not correlated to OS for all ovarian cancer patients HR, 0.92 (0.8–1.05), *p* = 0.2 (Figure 3A). However, *Notch3* mRNA high expression was significantly correlated to favorite PFS for all ovarian cancer patients, HR 0.78 (0.68–0.89), *p* = 0.00026 (Figure 3B). *Notch3* mRNA high expression was not correlated to PPS in ovarian cancer patients, HR 1.07 (0.9–1.28), *p* = 0.44 (Figure 3C).

**Figure 4:**
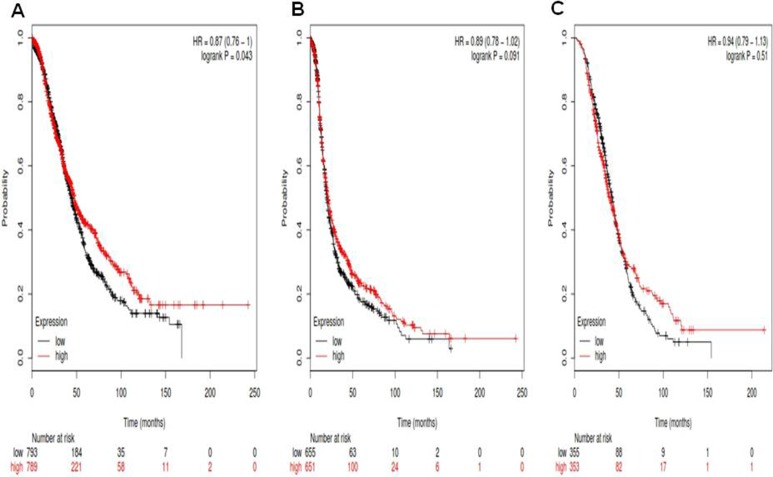
For Notch4, its Affymetrix ID is 205247_at (**A**) OS curves are plotted for all ovarian cancer patients (*n* = 1,582). (**B**) PFS curves are plotted for ovarian cancer patients (*n* = 1,306). (**C**) PPS curves are plotted for ovarian cancer patients (*n* = 708).

For Notch4, its Affymetrix ID is 205247_at. *Notch4* mRNA high expression was significantly correlated to favorite OS for all ovarian cancer patients, HR 0.87 (0.76–1), *p* = 0.043 (Figure 4A). *Notch4* mRNA high expression was not significantly correlated to PFS for all ovarian cancer patients, HR0.89 (0.78–1.02), *p* = 0.091 (Figure 4B). *Notch4* mRNA high expression was not correlated to PPS in ovarian cancer patients, HR 0.94 (0.79–1.13), *p* = 0.51 (Figure 4C).

For further access the correlation of individual Notch receptor mRNA expression with other clinicopathological features, we examined the correlation of PFS with different histological types (Table 1), pathological grades (Table 2), clinical grades (Table 3) and *TP53* mutation (Table 4) of ovarian cancer patients. As from Table 1, all the individual Notch receptors were not significantly associated with PFS in different histological types of ovarian cancer patients. In addition, all the individual Notch receptors were also not significantly associated with OS and PPS in different histological types of ovarian cancer patients (data not shown). From Table 2, all the individual Notch receptors except *Notch 2* were not significantly associated with PFS in pathological grades of ovarian cancer patients. *Notch 2* mRNA high expression was associated with poor PFS in grade II ovarian cancer patients, HR 1.45 (1.07–1.96), *p* = 0.016. In addition, *Notch 4* mRNA high expression was associated with favorite OS in grade III ovarian cancer patients, HR 0.76 (0.64–0.9), *p* = 0.0018. From Table 3, all the individual Notch receptors were not significantly associated with PFS in clinical stages of ovarian cancer patients. However, *Notch 1* mRNA high expression was associated with favorite OS in clinical stage III ovarian cancer patients, HR 0.83 (0.7–0.98), *p* = 0.03. *Notch 3* mRNA high expression was also associated with favorite OS in clinical stage III ovarian cancer patients, HR 0.83 (0.7–0.99), *p* = 0.037. From Table 4, only *Notch 1* mRNA high expression was significantly associated with poor PFS in *TP53* wild type ovarian cancer patients, HR 1.86 (1.05–3.31), *p* = 0.031, but *Notch 1* mRNA high expression was significantly associated with favorite PFS in *TP53* mutation type ovarian cancer patients, HR 0.78 (0.6–0.99), *p* = 0.045.

**Table 1:** Correlation of Notch receptor mRNA high expression with PFS in different histology of ovarian cancer patients

Notch receptors	histology	Cases	HR 95% CI	*P* value
Notch 1	serous	1019	0.99 (0.85–1.15)	0.9
	endometrioid	36	0.48 (0.17–1.33)	0.15
Notch 2	serous	1019	1.13 (0.97–1.32)	0.11
	endometrioid	36	1.35 (0.5–3.62)	0.55
Notch 3	serous	1019	1.06 (0.91–1.23)	0.45
	endometrioid	36	1.59 (0.58–4.38)	0.36
Notch 4	serous	1019	1.07 (0.92–1.25)	0.37
	endometrioid	36	0.69 (0.26–1.84)	0.45

**Table 2:** Correlation of Notch receptor mRNA high expression with PFS in pathological grades of ovarian cancer patients

Notch receptors	Pathological grades	Cases	HR 95% CI	*P* value
Notch 1	I	37	0.68 (0.23–2.03)	0.48
	II	247	1.19 (0.89–1.61)	0.25
	III	790	0.93 (0.78–1.11)	0.44
Notch 2	I	37	0.93 (0.31–2.77)	0.89
	II	247	1.45 (1.07–1.96)	0.016
	III	790	1.03 (0.87–1.23)	0.71
Notch 3	I	37	1.66 (0.54–5.08)	0.37
	II	247	1.21 (0.89–1.63)	0.22
	III	790	0.94 (0.79–1.12)	0.48
Notch 4	I	37	0.38 (0.12 −1.22)	0.091
	II	247	1.03 (0.76–1.38)	0.86
	III	790	1.04 (0.88–1.24)	0.64

Note: three-tier grading scheme for pathological classification.

**Table 3:** Correlation of Notch receptor mRNA high expression with PFS in clinical stages of ovarian cancer patients

Notch receptors	Clinical stages	Cases	HR 95% CI	*P* value
Notch 1	I + II III IV	126 846 143	0.99 (0.55–1.79) 0.98 (0.83–1.15) 1.05 (0.7–1.57)	0.97 0.80 0.81
Notch 2	I + II III IV	126 846 143	0.79 (0.43–1.43) 1.07 (0.91–1.26) 1.48 (0.98–2.22)	0.43 0.39 0.059
Notch 3	I + II III IV	126 846 143	1.09 (0.6–1.98) 0.99 (0.84–1.16) 1.01 (0.67–1.5)	0.77 0.86 0.98
Notch 4	I + II III IV	126 846 143	1.12 (0.61–2.06) 1.09 (0.93–1.28) 1.37 (0.92–2.06)	0.71 0.30 0.12

Note: four-tier grading scheme for clinical stages.

**Table 4:** Correlation of Notch receptor mRNA high expression with PFS in *TP53* mutation status of ovarian cancer patients

Notch receptors	*TP53* mutation	Cases	HR 95% CI	*P* value
Notch 1	No Yes	76 416	1.86 (1.05–3.31) 0.78 (0.6–0.99)	0.031 0.045
Notch 2	No Yes	76 416	0.84 (0.48–1.48) 1.05 (0.82–1.35)	0.55 0.68
Notch 3	No Yes	76 416	0.74 (0.42–1.31) 0.88 (0.69–1.13)	0.31 0.33
Notch 4	No Yes	76 416	1.15 (0.65–2.03) 0.94 (0.73–1.21)	0.63 0.64

## DISCUSSION

Notch1 was widely reported in ovarian carcinogenesis and was the best studied among Notch ligands and four Notch receptors [12–15]. The active form of Notch 1, the Notch 1 intracellular domain (NICD), was detected in ovarian cancer cell lines, ovarian cancer specimens and may led to growth inhibition of ovarian cancer cells upon depletion of Notch 1 by Notch 1 siRNA [42]. Down-regulation of Notch1 expression was significantly inhibit cell growth, induce G1 cell cycle arrest and induce cell apoptosis in A2780 ovarian cancer cells [43]. Notch1 NICD was reported to be an independently poor prognostic factor in ovarian cancer patients [44]. In this study, we found that *Notch1* mRNA high expression was not correlated to PFS for all ovarian cancer patients. However, *Notch 1* mRNA high expression is significantly associated with poor PFS in *TP53* wild type, but favorite PFS in *TP53* mutation type ovarian cancer patients. In addition, *Notch1* mRNA high expression was also not correlated to PFS ovarian cancer patients, 0.93 (0.81–1.06), *p* = 0.27, as well as PPS in ovarian cancer patients, HR 1.17 (0.98–1.4), *p* = 0.081.

Notch2 was aberrant expressed ovarian cancer cells [44]. These results indicate that Notch2 seems to be a tumor suppressor in ovarian carcinogenesis. In this study, we found that *Notch2* mRNA high expression was significantly correlated to poor PFS for all ovarian cancer patients, especially in grade II ovarian cancer patients. However, *Notch2* mRNA high expression was not correlated to poor PFS in serous and endometrioid cancer patients. In addition, *Notch2* mRNA high expression was not correlated to OS for all ovarian cancer patients HR, 0.96 (0.84–1.1), *p* = 0.54. *Notch2* mRNA high expression was also not correlated to PPS in ovarian cancer patients, HR 1.09 (0.91–1.31), *p* = 0.34.

Notch3 high protein expression was detected in high-grade ovarian tumors [45]. Inactivation of Notch3 suppressed cell proliferation and induced apoptosis in the ovarian cancer cells [45]. Jagged-1/Notch3 interaction constitutes a juxtacrine loop promoting proliferation in ovarian cancer cells [46]. Notch 3 protein overexpression was associated with ovarian cancer metastasis, chemoresistance and poor overall survival in ovarian serous cancer patients [47]. Inhibition of Notch3 inhibited ovarian cancer growth and induced apoptosis [48]. In comparison with gamma-secretase inhibitor (GSI) in the treatment of paclitaxel in paclitaxel-resistant cancer cells, Notch3 siRNA specific inhibition showed more efficacy [49]. This approach of using more specific individual Notch member inhibitor would be likely to avoid the side effects of broad-spectrum GSI treatment and has more potential to use in clinical setting. Our results showed that *Notch3* mRNA high expression was significantly correlated to favorite PFS for all ovarian cancer patients. However, *Notch3* mRNA high expression was not correlated to OS for all ovarian cancer patients HR, 0.92 (0.8–1.05), *p* = 0.2; *Notch3* mRNA high expression was also not correlated to PPS in ovarian cancer patients, HR 1.07 (0.9–1.28), *p* = 0.44.

Notch4 was reported as an oncogene in mammary carcinogenesis [50–51]. Notch4 significantly increased the tumorigenic potential [52–53]. Gao et al. [54] reported that Notch4 may be also an oncogene in ovarian carcinogenesis, since Notch4 was involved in modulating many functions of stem cells. We found that *Notch4* mRNA high expression was not significantly correlated to PFS for all ovarian cancer patients. However, *Notch4* mRNA high expression was significantly correlated to favorite OS for all ovarian cancer patients, HR 0.87 (0.76–1), *p* = 0.043. *Notch4* mRNA high expression was not correlated to PPS in ovarian cancer patients, HR 0.94 (0.79–1.13), *p* = 0.51.

Notch members and TP53 are gene transcription regulators that are critically involved in various aspects of stem cell maintenance, cell differentiation, and tumor progression. Thus, extensive crosstalks between the Notch and TP53 pathways were reported about above processes [55]. TP53 and some of Notch members have also been identified as potential prognostic biomarkers in ovarian cancer patients [47, 56–57], however, there are no report about the association between TP53 and Notch members in ovarian cancer. Interestingly, there are strong evidences showing the correlation between TP53 and Notch members in breast cancer [58]. In our analysis, only *Notch 1* mRNA high expression was significantly associated with poor PFS in *TP53* wild type ovarian cancer patients, HR 1.86 (1.05–3.31), *p* = 0.031, but *Notch 1* mRNA high expression was significantly associated with favorite PFS in *TP53* mutation type ovarian cancer patients, HR 0.78 (0.6–0.99), *p* = 0.045. These results indicate that *TP53* status significantly impact the prognostic value of *Notch 1* in ovarian patients.

Previous results suggest that Notch signaling, especially Notch receptors may be essential drug target for ovarian cancer patients. However, so far, not many specific small molecular inhibitors or other antagonists of the different Notch members have been developed. γ-secretase inhibitor, DAPT was demonstrated to inhibit Notch activation and cell growth in ovarian cancer cells [21]. However, γ-secretase inhibitors are not able to distinguish individual Notch receptors and may cause intestinal toxicity [59] by inhibiting other signaling pathways [60]. Recently, highly specialized antibodies which can recognize each Notch receptor paralogue were developed by phage display technology in human patients and rodent models [61]. Based on our study that *Notch1* mRNA high expression was significantly correlated with poor PFS in *TP53* wild type ovarian cancer patients. *Notch2* mRNA high expression was significantly correlated with poor PFS for all ovarian cancer patients, especially in grade II patients. Thus Notch1 and Notch2 might be potential drug targets for some types of ovarian cancer patients.

In summary, we demonstrated that *Notch 1* mRNA high expression is significantly associated with poor PFS in *TP53* wild type, but favorite PFS in *TP53* mutation type ovarian cancer patients. *Notch2* mRNA high expression was significantly correlated to poor PFS for all ovarian cancer patients, especially in grade II ovarian cancer patients. *Notch3* mRNA high expression was significantly correlated to favorite PFS for all ovarian cancer patients. *Notch4* mRNA high expression was not significantly correlated to PFS for all ovarian cancer patients. These results will be useful for favorite understand the heterogeneity and complexity in the molecular biology of ovarian cancer and to develop tools to more accurately predict their prognosis.

## MATERIALS AND METHODS

KM plotter was used to analyze the correlation of individual Notch receptor mRNA expression to overall survival (OS), progression-free survival (PFS), as well as post-progression survival (PPS). The background database include lung cancer [26], breast cancer [24], gastric cancer, as well as ovarian cancer [25] database. Ovarian cancer patients in the database were identified from Cancer Biomedical Informatics Grid (caBIG, https://biospecimens.cancer.gov/relatedinitiatives/overview/caBig.asp), the Gene Expression Omnibus (GEO, http://www.ncbi.nlm.nih.gov/geo/) and The Cancer Genome Atlas (TCGA, https://cancergenome.nih.gov/) ovarian cancer datasets [25]. They contain clinical data such as gender, age, histology, grade, stage, applied chemotherapy and *TP53* mutation status for all patients in WinStat 2013. The ovarian cancer patients were followed up 20 years. The database collected survival information of 1,648 ovarian cancer patients downloaded from Gene Expression Omnibus (GEO). Four Notch sub-members (*Notch1~4*) were put into the database (http://kmplot.com/analysis/index.php?p=service&cancer=ovar) to obtain Kaplan-Meier various survival plots. In order to determine the prognostic value of a particular gene, the samples were split into two groups according to the median expression of the gene. The certain gene mRNA expression above or below the median separates the cases into high expression and low expression. KM plotter also provides options to split patients by lower quartile, lower tertile, upper tertile, upper quartile expression, but only median expression giving almost same numbers of two groups and less biasing. Hazard ratio (HR), 95% confidence intervals and log rank P were analyzed and presented on the main plots. *P* value of < 0.05 was considered to be statistically significant. HR is the ratio of the hazard rates corresponding to the conditions described by two levels of an explanatory variable in survival analysis.
